# Education Research: The Neurohumanities in Training: Integrating a Humanities Curriculum Within Neurology Residency Programs

**DOI:** 10.1212/NE9.0000000000200178

**Published:** 2024-11-14

**Authors:** Mattia Rosso, Tatiana Greige, Charles Palmer, Greta Solinap Peng, Rumyar V. Ardakani, Alexander Frolov, Manju George, Raphael Arellano Carandang, Galina Gheihman, Michael P.H. Stanley

**Affiliations:** From the Department of Neurology (M.R., C.P.), Medical University of South Carolina, Charleston; Department of Neurology (T.G.), Boston Medical Center, MA; Department of Neurology (G.S.P.), University of California San Francisco; Department of Neurology (R.V.A.), University of Colorado Anschutz Medical Campus, Aurora; Department of Neurology (A.F., M.G.), The University of Texas Southwestern Medical Center, Dallas; Department of Neurology (R.A.C.), University of Massachusetts Medical School, Worcester; Mass General Brigham Neurology Residency Program (G.G.), Brigham and Women's Hospital and Massachusetts General Hospital, Boston; and Neurocognitive Division (M.P.H.S.), Tufts Medical Center, Boston.

## Abstract

**Background and Objectives:**

Perhaps stemming from the central role of detailed examinations and a focus on the subjective sphere that grounds their clinical practice, neurologists have frequently opined on experiences traditionally a province of humanities. The increasingly technological focus on medical education and care can be seen to devalue the subjective aspects of medicine. As a counter to this, we report on the existence of neurohumanities curricula within neurology residency training.

**Methods:**

We conducted an exploratory descriptive analysis of a convenience sample of 6 neurology residency programs in the United States with neurohumanities curricula. We reported the objectives of each program and feedback from participants. Finally, we described and identified patterns within the curricula and participant feedback.

**Results:**

A shared feature of all programs was recency because all were started within the past decade. Seven sources of variability were timing, target audience, setting, scope of didactics, funding, regional differences, and objectives. The events ranged from mandatory to optional, from fully integrated in residency didactics to extracurricular. While residents were the primary audience across all programs, medical students and faculty were included as optional in some of the curricula. Objectives varied from clinical skill enhancement (e.g., improving observation through art), wellness (e.g., narrative medicine, self-reflection), to the scholarly exploration of the intersection between humanities and neuroscience.

**Discussion:**

Our findings illustrated different ways of integrating humanities into neurology residency training. We highlighted the diverse approaches and objectives adopted by each program, which ranged from pedagogy to wellness. We hope this preliminary study will serve as a first step in the broader assessment of the needs, which neurohumanities curricula can address within neurology training. We also hope that this will lead to more formal assessment of the possible benefits of such implementation, which may include reflecting on clinical practice, debriefing from stressful events, and engaging with humanities.

## Introduction

William Osler stated that medicine is an art based in science, “a calling in which your heart will be exercised equally with your head.”^1^ The degree to which this has remained the case in medical training has changed over time, and several attempts have been made to instill more exposure to humanities.^2^ At the undergraduate medical education level, humanities curricula are common, although there have been criticisms regarding a lack of standardization.^3-5^ More recently, the Association of American Medical Colleges has put forth the Fundamental Role of Arts and Humanities in Medical Education initiative, which attempts to provide guidelines and toolkits for the implementation of humanities curricula within medical schools.^3^ Within medical schools and medical practice, the intersection of the humanities, art, and medicine has been a means to address and frame questions about the meaning of illness, providing more holistic avenues to the practice of medicine.^4,6-10^ This common sense belief is supported by research, which has shown improved observational skills and empathy among medical students after exposure to humanities.^11,12^ Interest in humanities within medicine is counterbalanced by the impact of societal, scientific, and technological changes that have altered the patient-physician relationship, at times distancing physicians from their patients.^13-16^ Among other changes, the use of electronic medical records has contributed to a shift in the traditional patient-physician dynamic.^17,18^

Within neurology, the interest in humanities has been exemplified by important authors such as Andrew Lees, Oliver Sacks, and Alexander Luria, to name a few. Each of these authors illustrated the links between neuroscience and works of literature and music, the importance of a phenomenologic approach to neuroscience, and the centrality of the patient's subjective narratives within neurology.^19-24^ Some of these works can be considered exercises in neurohumanities, although this term has taken different connotations over the years. These have included the study of the humanities of neurology, a neuroscientific approach to humanities, neuroaesthetics, and a humanistic approach to neuroscience.^24,25^ One definition of “neurohumanities” highlighted the value of studying human experience (including art, literature, and philosophy) through a neuroscientific framework.^26^

Similar efforts are seen in neurology residency programs, where art-based, narrative medicine, and neuroethics curricula have been introduced.^27-30^ However, no comprehensive case series on well-rounded neurohumanities curricula has been published in academic journals. Partially covered in a recent media report, we present a preliminary assessment of 6 neurohumanities curricula within neurology residency training.^31^ In this article, we will describe the origins, rationale, objectives, pedagogical approaches, and outcomes of these programs. Finally, we will conclude by discussing research gaps and suggested future directions for neurohumanities in the context of neurology residency training.

## Methods

### Study Team/Reflexivity Statement

The authors of this study consist of a multidisciplinary team of neurology residents, fellows, and attending physicians with a shared interest in neurohumanities and the intersection between neurology and arts. Driven by our own personal experiences in clinical practice, we recognized the need to complement traditional neurology training with initiatives that promote and incorporate neurohumanities with the aim to provide a holistic approach to residency education, promote empathy, and provide a safe space for self-reflection. We wished to gain an understanding of the availability of such programs across residency programs and the nature of these programs and began with an exploratory, descriptive preliminary review.

### Study Design and Participant Selection

Convenience sampling of US-based neurology residency programs was used to identify programs with neurohumanities curricula. The sampling process leveraged personal and working contacts of the authors, contacts through humanities-related professional societies (e.g., the American Osler Society, the American Academy of Neurology [AAN]), and social media outreach (through X, formerly known as Twitter). The authors also conducted a systematic search of the internet to identify any other potential participants. With the assistance of an academic librarian, the databases PubMed, SCOPUS, and Google Search were searched on February 6, 2024, across all available date ranges using the following keywords in combination or alone: “neurohumanities,” “neurology residency humanities program,” “neuroscience AND humanities,” “(humanities OR philosophy OR arts OR ethics OR social sciences) AND (neuroscience OR neurohumanities),” and “art in neurology residency.” In addition, programs highlighted in *Neurology Today*'s coverage on leading humanities programs were invited to join.^31^ Six neurology residency programs in the United States with a neurohumanities curriculum were identified, although none were identified through a search engine or database.

### Survey Design and Distribution

The authors distributed a nine-question survey to gather descriptive information from each neurohumanities program (see eTable 1 for full survey), specifically asking the following relevant features of each program: starting year, leadership make-up, scope, target participants, and funding resources. The survey was developed by M.R. and C.P. using Microsoft Word (version 16.71; Microsoft, Seattle, WA, 2023). The survey was distributed through email to the organizers of the previously identified neurohumanities programs. Participants were asked to provide written responses. Participation was voluntary. By agreeing to respond to the survey, each participating institution granted permission for the reporting of their respective program's characteristics and curricula.

### Feedback Collection

Each institution also used its own approach to gather feedback about the programs. The authors asked survey respondents to provide sample, anonymized feedback from local participants about their programs through optional, unstructured free-form writing. Participants at each institution were informed that their written feedback would be used for research purposes and that their answers would be anonymized and deidentified.

### Data Analysis

We completed an exploratory descriptive analysis of the various neurohumanities curricula implemented within multiple neurology residency programs across the United States. The primary goal was to provide a preliminary overview of the different neurohumanities initiatives, including their structure, implementation, and perceived effectiveness and impact. Qualitative data from survey responses and open-ended feedback responses were reviewed to identify common objectives across the different neurohumanities programs and explore the experiences and perspectives of the participants on integrating a neurohumanities curriculum in neurology residency. As a case series, we treated each program as a case and summarized the key features in Tables 1–3. Commonalities and variability between the cases were then identified and described to show the distribution of variability. Finally, we described and summarized the feedback from program participants provided by each survey respondent. The summary was developed by several of the authors including M.R., C.P., G.G., and T.G. The summary was then submitted to the rest of the authors and agreed upon. The purpose of this analysis was to understand what outcomes were achieved by the local neurohumanities initiatives, as perceived by program participants.

**Table 1:** Summary of Basic Demographics of the Neurohumanities Programs and Hosting Institutions

Name	Geographical location	Initial year	Summary of program structure and aims
BUMC (MANET* project)	Boston, MA	2022	Collaboration with the Harvard Art Museums: Multiple in-person sessions at Fogg Museum Focuses on visual arts
MGB	Boston, MA	2023	Arts and humanities programming offered every other month during daily formal didactics Sessions have included poetry reading and analysis, music appreciation, and arts and crafts
MUSC	Charleston, SC	2021	Monthly meeting with in-person or virtual discussions Mix of experiential events (museum visit, film viewing) and seminar-based
UCSF	San Francisco, CA	2022	Sessions that engage residents in narrative medicine (i.e., creative writing) or reflections on artwork (literature, audio, visual) Writing buddies: pairing of residents with feedback on written narrative medicine pieces
UMASS	Worcester, MA	2017	Monthly 1-h session of invited speakers on curated topics that explore the interaction of neuroscience and art, music and literature, re-emphasize humanistic practice of neurology and encourage self-reflection and foster self-awareness
UTSW	Dallas, TX	2022	Lectures, seminars, and group discussions on humanism in neurology, narrative medicine, philosophy of mind, history of neurology, and practice of neurology External activities (i.e., museum visits) Optional after-hours off-campus get-togethers for smaller group discussion of interesting works

Abbreviations: BUMC = Boston University Medical Center; MANET* = The Museum Art in Neurology Education Training; MGB = Mass General Brigham Combined Program; MUSC = Medical University of South Carolina; UCSF = University of California, San Francisco; UMASS = University of Massachusetts Medical School; UTSW = University of Texas Southwestern.

**Table 2:** Program Characteristics in Detail

Program	Timing	Intended audience	Leadership	Frequency of meeting	Funding sources
BUMC (MANET* project)	Didactics, work hours	Residents Open to medical students, faculty	Resident-led	Quarterly (3–4 sessions per year)	Departmental funding
UCSF	After hours	Residents	Resident-led	Every 2 months (5–6 sessions per year)	Residency wellness fund
MGB	Noon conference	Residents Open to medical students, faculty	Resident-led	Every 2 months (5–6 sessions per year)	Education
MUSC	After hours	All comers	Resident-led	Monthly	Departmental funding
UMASS	Noon lecture	Residents but open to students, fellows and faculty	Program director/faculty led	Monthly	Department funding
UTSW	Academic half-day After hours	Residents	Faculty led	Monthly	Departmental funding

Abbreviations: BUMC = Boston University Medical Center; MANET* = The Museum Art in Neurology Education Training; MGB = Mass General Brigham Combined Program; MUSC = Medical University of South Carolina; UCSF = University of California, San Francisco; UMASS = University of Massachusetts Medical School; UTSW = University of Texas Southwestern.

**Table 3:** Program Objectives

Program	Objectives
BUMC (MANET* project)	To improve observational skills To enhance residents' empathy and tolerance for ambiguity To provide residents with a space for self-reflection and expression
UCSF	To engage in narrative medicine with coresidents and faculty To provide spaces for self-reflection
MGB	To reflect, engage, and foster wellness and well-being through arts and humanities
MUSC	To build a space for trainees to explore the intersection of neurology and the humanities To explore work emphasizing the neuroscientific aspects of the arts and the humanities
UMASS	To re-emphasize the humanistic aspects of medical practice To improve self-awareness of how our perspectives and emotions influence our practice To explore the intersection of art and neurology To provide a positive outlook on the impact of neurology on the world To obviate burnout
UTSW	To foster and strengthen the connection between neurologists and their patients by harnessing key concepts of the medical humanities

Abbreviations: BUMC = Boston University Medical Center; MANET* = The Museum Art in Neurology Education Training; MGB = Mass General Brigham Combined Program; MUSC = Medical University of South Carolina; UCSF = University of California, San Francisco; UMASS = University of Massachusetts Medical School; UTSW = University of Texas Southwestern.

### Standard Protocol Approvals, Registrations, and Patient Consents

Institutional review board approval was not sought because this study was not human subjects research. The data involved the collection of program evaluation information about the neurohumanities programs and not individual perspectives from the program directors.

### Data Availability

Anonymized data not published within this article will be made available by request from any qualified investigator.

## Results

Table 1 outlines the demographics and geographic location of each program included in our sample. Table 2 presents the characteristics of each programs' curriculum, as extracted from our survey. We described and summarized the distribution in the 6 case programs in the following 7 key characteristics: (1) timing, (2) intended audience, (3) setting, (4) didactic scope, (5) sources of funding, (6) regional distribution, and (7) the program's objectives. Table 3 details the objectives of each program. The response rate was 100% among the identified groups. Below we discuss each of these in further detail and provide sample examples from our cases.

### Timing

Four programs (Boston University Medical Center [BUMC], Mass General Brigham Combined Program [MGB], University of Texas Southwestern [UTSW], University of Massachusetts Medical School [UMASS]) implemented humanities as a core component of their annual residency curriculum, which was mandatory for all residents. Two of these (BUMC and UTSW) held their meetings during academic half-days, whereas the other 2 (MGB, UMASS) featured it as part of noon lecture within the rotating curriculum. The MGB program was featured as part of the “Well-Rounded Wednesdays” series, which would take place on the second Thursday of the month. Two programs (Medical University of South Carolina [MUSC] and University of California, San Francisco [UCSF]) organized an extracurricular humanities series, which was held after working hours on a nonmandatory basis. Of note, UTSW also recently initiated a separate extracurricular neurohumanities series, which was also nonmandatory.

### Intended Audience

The target audience was a second source of variability. Residents comprised the primary audience for the UCSF and UTSW groups. A wider audience (i.e., the entire neurology department) was selected by the BUMC, MGB, and UMASS groups. The MUSC group was open to all interested parties, including MUSC clinicians, members of other institutions, and others (Table 2).

### Setting

We also observed significant differences in settings, ranging from fully in-person to hybrid. In-person events were favored by the BUMC and UTSW. MGB and UCSF were in-person with additional remote options, whereas MUSC was predominantly hybrid, allowing both in-person and remote viewing. UMASS started as in-person but moved to hybrid during the coronavirus disease pandemic and has continued in that format.

### Didactic Scope

The didactic scope ranged from a narrow focus (e.g., narrative medicine, art) to a broader scope (e.g., a rotating curriculum of speakers). Museum-based events were seen in the BUMC, MUSC, and UTSW programs. These events are designed to focus on the parallels between clinical observation and visual arts. UCSF developed resident-led events with 2 components, that is, narrative medicine and a parallel “writing buddies” program. The narrative medicine series was built around a reflection on the practice of medicine through different media, including writing, visual arts, and audio media (Table 1). MGB included sessions on neurohumanities subjects as part of a larger wellness curriculum, which included poetry reading and analysis, music appreciation, arts and crafts, and journaling. MUSC developed a mix of experiential events (museum visits, coffee tasting, wine and music nights) along with formal discussion-based seminars (e.g., “Rodin and Charcot,” “Medical Humanities in the Age of AI,” and “The Doctor's Note as a short story”). The UMASS curriculum was designed around core topics that explored the intersection of neuroscience and humanities, including art in neuroscience, history of neurology, and narrative medicine. This curriculum also benefitted from the exploration of lesser known topics, such as the neuroscience of magic.

### Sources of Funding

Programs differed on funding sources, which included funding under educational, departmental, or wellness budgets. Educational funds were primarily used by programs held as part of the existing didactic curriculum, such as in the case of MGB. Wellness funds were used by UCSF, given the after-hours setting. Departmental funds were used by the remainder.

### Regional Distribution

The surveyed programs encompassed a wide range of regions in the United States with 3 programs from the Northeast, 2 from the South, and 1 from the Pacific Northwest (Table 1).

### Objectives

The programs also showed a remarkable variability in their objectives. Four patterns transpired and included (1) the exploration of the intersection of neurology and humanities (e.g., the neuroscientific underpinnings of art and literature in the practice of medicine), (2) art as a source of betterment of clinical skills (e.g., observational skills through museum visits), (3) narrative medicine (e.g., humanities as a source of self-reflection, self-expression, connection with patients, awareness of the art of medicine), and (4) humanities as a source of wellness and antidote to burnout (Table 3).

Regarding collected feedback, 4 concepts or outcomes were identified as being emphasized within the neurohumanities program. It was felt by participants across all the programs that neurohumanities initiatives (1) enhanced empathy and improved communication and observational skills; (2) improved self-awareness and provided opportunity for self-reflection; (3) fostered well-being and improved camaraderie amongst trainees; and (4) explored the intersection between neurology, medicine, and humanities. We selected specific excerpts from participant feedback as examples to define and illustrate each core concept (Table 4).

**Table 4:** Sample of Collected Feedback From Different Institutions

Category and description	Feedback example	Institution
Establishing meaningful connections with patients To enhance empathy and improve communication and observational skills	Every meeting, we explore a different aspect of what it means to experience the world as a human, through the lens of neurology, and it helps me feel more connected to my patients, my colleagues and my own inner love for neurology	MUSC resident
	I also think that seeing artistic depictions of the human condition, in whatever form, is helpful to maintaining empathy. It's sometimes hard to see patients as a “whole.” These exercises force you to see a work of art both as its smaller details and components and as a whole, which we should be doing with patients!	BUMC resident
	Has the program impacted the way you see your role as a doctor, the way you see patients, the way you see colleagues, and/or the way you approach patient care? Yes. I'm more self-reflective and see my patients as persons more and that is reflected in the time I spend at the bedside and my motivation to see patients	UMASS resident
Exploring the intersection of neurology and humanities To reconnect with previous artistic interests and to reflect on how these interests may help shape one's current practice	I became a neurologist because I felt it was the field where I could be a sort of artist in my own way, and the neurohumanities program at MUSC has been the place where this has felt most possible and supported	MUSC resident
	I think the session I attended was really helpful for me to improve my observational skills and to think about different perspectives and angles of the work, which I have applied to neurology as I have been using those skills to come up with differentials for new cases	BUMC resident
Establishing meaningful connections with fellow colleagues To foster well-being and improving camaraderie and teamwork among trainees	I am an individual who became interested in medicine by way of the arts, and I've often felt the algorithmic approach to practicing medicine falls short. This year the group has participated in a variety of activities that I believe have helped strengthen a different perspective	MUSC resident
Improving self-awareness and providing opportunity for self-reflection To deepen one's appreciation for neurology by stepping away from the “grind” of residency and to perceive their dedication to patient care in a more humanistic way	Having this space has reduced burn out and given me an outlet to see the bigger picture of practicing neurology in a human facet. Rather than “resilience training” this humanities group represents an effective solution to engaging other interests and refreshing the overworked resident soul	MUSC resident
	Our Neurohumanities group has permitted my intellectual curiosity to blossom and has deepened my intrigue for neurosciences while allowing me to apply these new concepts and knowledge into my daily medical practice with real life patients	MUSC resident

Abbreviations: BUMC = Boston University Medical Center; MANET* = The Museum Art in Neurology Education Training; MGB = Mass General Brigham Combined Program; MUSC = Medical University of South Carolina; UCSF = University of California, San Francisco; UMASS = University of Massachusetts Medical School; UTSW = University of Texas Southwestern.

## Discussion

In this article, we described the implementation of 6 neurohumanities curricula within neurology residency training. We started with a convenience sample of the neurohumanities curricula featured in a recent media report. To this list, we added additional curricula from a convenience sample of the authors' networks. We examined these through a descriptive analysis, summarizing 7 key characteristics of each curriculum. These included (1) timing, (2) intended audience, (3) setting, (4) didactic scope, (5) sources of funding, (6) regional distribution, and (7) program's objectives.

We observed diversity in timing and settings, ranging from mandatory events during the workday to optional events held after hours. This also determined the nature of the audience. For instance, resident-led, small group sessions were favored for more intimate exchanges of narrative medicine (e.g., UCSF), whereas larger hybrid events were conducive to more formal seminars (e.g., MUSC). This diversity also aligned with the didactic scope—for instance, mandatory curricula tended to focus on pedagogy. An example of this would be curricula that used art-focused museum events as a tool to enhance clinical observation. On the other hand, programs with optional, after-hours curricula tended to focus on wellness or narrative medicine. These results show that neurohumanities curricula can be integrated in a multitude of ways into neurology residency. An important commonality of these programs was their recent start date within the past decade, which we speculate may be related to the more recent changes in medical practice. While attempts to re-emphasize the role of humanities in medical education date back to the 19th and 20th centuries, the threat of dehumanization has become even more pronounced in recent decades.^2^ Today, the rise in medical technologies, including electronic medical records and advanced neuroimaging,^13-16^ along with bureaucratic demands for cost-effectiveness, time efficiency, and codified notetaking have contributed to distancing clinicians from the bedside. While these changes offer certain benefits, they have also been implicated as threats to the patient-physician relationship, potentially leading to the objectification and dehumanization of patients.^17,18,32,33^

Humanities have gained a growing recognition within undergraduate medical education with the development of important curricula, which have included Dr. Remen's *Healer's Art* curriculum, several different curricula focused on Rita Charon's work on narrative medicine, and art-based curricula.^34-37^ Nonetheless, our article adds 2 important dimensions because these newly described curricula are set within residency training and with a focus on neurohumanities specifically. Despite the busy schedules of neurology trainees, we showed that these curricula are feasible and beneficial. The positive feedback from participants on increased connection with their patients, with other participants, and with their own artistic interests is particularly remarkable in an era of heightened physician burnout. The option of remote participation (e.g., using teleconferencing software), whose use has expanded in the wake of the severe acute respiratory syndrome coronavirus 2 pandemic, facilitated attendance especially at after-hours events.

Our study has several limitations. We relied on convenience sampling and the use of unstructured, open-ended questions for feedback from participants and program leaders. The initial identification of potential participants relied on multiple mechanisms including social media, personal and research connections, as well as a literature review of prior published initiatives. Although convenience sampling allowed for a targeted approach given the exploratory nature of this study, our query may have missed ongoing neurohumanities initiatives without published information. This introduces a risk of sampling bias and limits the generalizability of our findings. Furthermore, while we discussed neurohumanities in training as a recent phenomenon, it is possible that past iterations of such efforts may have gone unpublished. A second limitation is the use of a retrospective survey, in which we relied on self-reported data from program organizers. This is subject to response bias. Feedback from program participants was also gathered using unstructured, nonstandardized, open-ended questions. Although acknowledging that the heterogeneity in the descriptions may have introduced reporting bias, we felt that unstructured, open-ended feedback was more suited for rich descriptions from curriculum participants.

Despite its limitations, our study provides valuable insights into implementing neurohumanities curricula within neurology residency training. A key strength of the study is the use of participant feedback, which provides a valuable qualitative and personal perspective on these initiatives and their benefits. The diversity of approaches explored also offers potential avenues for future efforts. Our results align with earlier reports on increased interest in neurohumanistic endeavors, particularly those examining neuroethics curricula,^38^ as well as art-based and narrative medicine curricula, where participants noted improved awareness of diverse perspectives, increased self-reflection, and greater appreciation of their colleagues.^28^

Future directions would include a more comprehensive outreach to ensure a representative sample of neurohumanities curricula. As shown in our article, the spectrum of these curricula range from medical education to wellness and to original scholarly work in the field of the humanities. As such, we believe that each of these goals deserves its own pathway within neurohumanities. Within each pathway, we would advise setting standards and best practices. For instance, medical education–focused neurohumanities pathways may benefit from establishing learning goals, competencies, and expected outcomes. On the other hand, wellness-focused neurohumanities may benefit from more formal thematic analysis of the feedback and experiences of participants. Finally, a scholarly pathway may blossom by linking such curricula with other neurohumanities groups based within other areas of academia (e.g., philosophy departments, neuroscience departments, and so forth). Other neurohumanities-focused groups include the Neurohumanities research group at Duke University; the Neuro Humanities Studies Network; and the Art, Humanities, and Neuroscience Fellowships program at the Italian Academy.^39^ This would allow trainees to find key mentors to produce original scholarly work and potentially set up a successful career within this field. Each pathway would then benefit from an accurate and systematic analysis of the curricula implemented, the participants engaged within the pathway, and the goals achieved.

Finally, interested programs may seek financial and institutional support to expand program outreach. Depending on the program's scope and objectives, organizers might seek backing from institutions such as the AAN, American Neurological Association, or Accreditation Council for Graduate Medical Education. The AAN Annual Meeting has featured seminars on nonclinical topics, including wellness, music, and arts, which could be further expanded to include neurohumanities. This support could be crucial in broadening the reach of neurohumanities and in securing funding for future endeavors and related scholarship.

Our article provided the first case series of neurohumanities curricula within neurology residency training. While separate endeavors had been individually described, our article showcased several ways of using such curricula for the benefit of trainees ranging from pedagogy to wellness, as well as their positive impact on trainees. We hope our preliminary study will inspire interested residency programs and lead toward a comprehensive, systematic analysis of these curricula. We also advise interested groups to conduct formal surveys and assessments on their participants to estimate the impact of such programs. To do this, we suggest parsing out curricula into specific pathways based on the specific settings and goals of the groups. This approach will be crucial to identify best practices, suggest future directions, and perform a systematic evaluation of the benefits of neurohumanities within neurology residency.

## Appendix Authors

**Table:**

Name	Location	Contribution
Mattia Rosso	Department of Neurology, Medical University of South Carolina	Drafting/revision of the manuscript for content, including medical writing for content; major role in the acquisition of data; study concept or design; analysis or interpretation of data
Tatiana Greige	Department of Neurology, Boston Medical Center	Drafting/revision of the manuscript for content, including medical writing for content; major role in the acquisition of data; study concept or design; analysis or interpretation of data
Charles Palmer	Department of Neurology, Medical University of South Carolina	Drafting/revision of the manuscript for content, including medical writing for content; major role in the acquisition of data; study concept or design; analysis or interpretation of data
Greta Solinap Peng	Department of Neurology, University of California San Francisco	Drafting/revision of the manuscript for content, including medical writing for content; major role in the acquisition of data; study concept or design; analysis or interpretation of data
Rumyar V. Ardakani	Department of Neurology, University of Colorado Anschutz Medical Campus	Drafting/revision of the manuscript for content, including medical writing for content; major role in the acquisition of data; study concept or design; analysis or interpretation of data
Alexander Frolov	Department of Neurology, The University of Texas Southwestern Medical Center	Drafting/revision of the manuscript for content, including medical writing for content; major role in the acquisition of data; analysis or interpretation of data
Manju George	Department of Neurology, The University of Texas Southwestern Medical Center	Drafting/revision of the manuscript for content, including medical writing for content; analysis or interpretation of data
Raphael Arellano Carandang	Department of Neurology, University of Massachusetts Medical School	Drafting/revision of the manuscript for content, including medical writing for content; major role in the acquisition of data; study concept or design; analysis or interpretation of data
Galina Gheihman	Mass General Brigham Neurology Residency Program, Brigham and Women's Hospital and Massachusetts General Hospital	Drafting/revision of the manuscript for content, including medical writing for content; major role in the acquisition of data; study concept or design; analysis or interpretation of data
Michael P.H. Stanley	Neurocognitive Division, Tufts Medical Center	Drafting/revision of the manuscript for content, including medical writing for content; major role in the acquisition of data; study concept or design; analysis or interpretation of data

## Glossary

AAN: American Academy of Neurology
BUMC: Boston University Medical Center
MGB: Mass General Brigham Combined Program
MUSC: Medical University of South Carolina
UCSF: University of California, San Francisco
UMASS: University of Massachusetts Medical School
UTSW: University of Texas Southwestern
